# Why is it hard to make progress in assessing children’s decision-making competence?

**DOI:** 10.1186/1472-6939-16-1

**Published:** 2015-01-10

**Authors:** Irma M Hein, Pieter W Troost, Alice Broersma, Martine C de Vries, Joost G Daams, Ramón J L Lindauer

**Affiliations:** Department of Child and Adolescent Psychiatry, Academic Medical Center Amsterdam, Meibergdreef 5, 1105 Amsterdam, AZ The Netherlands; Department of Legal Affairs, Accare Child and Adolescent Psychiatry, PO Box 39, 9400 Assen, AA The Netherlands; Department of Medical Ethics and Health Law, Leiden University Medical Center, PO Box 9600, 2300 RC Leiden, The Netherlands; Department of Clinical Library, Academic Medical Center Amsterdam, Meibergdreef 9, 1105 Amsterdam, AZ The Netherlands

**Keywords:** Mental competence, Informed consent, Minor, Decision-making, Clinical research, Treatment, Assessment

## Abstract

**Background:**

For decades, the discussion on children’s competence to consent to medical issues has concentrated around normative concerns, with little progress in clinical practices. Decision-making competence is an important condition in the informed consent model. In pediatrics, clinicians need to strike a proper balance in order to both protect children’s interests when they are not fully able to do so themselves and to respect their autonomy when they are. Children’s competence to consent, however, is currently not assessed in a standardized way. Moreover, the correlation between competence to give informed consent and age in children has never been systematically investigated, nor do we know which factors exactly contribute to children’s competence.

This article aims at identifying these gaps in knowledge and suggests options for dealing with the obstacles in empirical research in order to advance policies and practices regarding children’s medical decision-making competence.

**Discussion:**

Understanding children’s competency is hampered by the law. Legislative regulations concerning competency are established on a strong presumption that persons older than a certain age are competent, whereas younger persons are not. Furthermore, a number of contextual factors are believed to be of influence on a child’s decision-making competence: the developmental stage of children, the influence of parents and peers, the quality of information provision, life experience, the type of medical decision, and so on. Ostensibly, these diverse and extensive barriers hinder any form of advancement in this conflicted area. Addressing these obstacles encourages the discussion on children’s competency, in which the most prominent question concerns the lack of a clear operationalization of children’s competence to consent. Empirical data are needed to substantiate the discussion.

**Summary:**

The empirical approach offers an opportunity to give direction to the debate. Recommendations for future research include: studying a standardized assessment instrument covering all four relevant dimensions of competence (understanding, reasoning, appreciation, expressing a choice), including a study population of children covering the full age range of 7 to 18 years, improving information provision, and assessing relevant contextual data.

## Background

The informed consent model assigns patients autonomy over medical interventions, including self-determination, with regard to their body, health, private life and sometimes even death. Exercising this autonomy regularly imposes a considerable burden of responsibility on the patient. However, where children and adolescents are concerned, we do not know if they are able to decide on medical issues in a meaningful way. There is no consensus on when they are competent to consent to treatment or clinical research.

Competence, as used it in this article, is the clinical concept of the ability of a person to consent to medical interventions or clinical research. Strictly speaking, incompetence denotes a legal status that in principle should be determined by a court. In clinical practice competence is generally addressed as decision-making capacity [1]. In this article we use the terms competence and decision-making capacity interchangeably, unless otherwise specified we are referring to the clinical assessment of capacity [1]. In adults, the generally accepted reference standard for competence assessment by clinicians revolves around four relevant criteria: to communicate a choice, to understand the relevant information, to appreciate the medical consequences of the situation, and to reason about treatment choices [2]. We are interested in children’s competence to consent in both the treatment context and the clinical research context, although these two may not be the same.

Although laws differ between nations and states, most laws prescribe that children and adolescents, hereinafter referred to as children, can exercise their rights as a patient from a certain age, including the right to give informed consent. However, these age limits vary considerably between countries and states.

Within the context of daily pediatric practice, competence is usually assessed implicitly. However, competency can become problematic when concerns rise about the capacities of pediatric patients to make well-considered decisions. For example, in some countries, when a 12-year old boy with acute leukemia is asked to participate in a drug trial, the researcher has the authority to judge the boy competent and his decision to consent to research participation valid or invalid. Or, in the case of a 15-year old girl with anorexia nervosa who, against her parents’ wishes, refuses to be tube fed, the treating pediatrician could be tasked with judging whether the girl is competent to refuse the proposed treatment. Again, this all depends on the local laws and regulations of the countries in which these cases occur.

For clinicians it is vital to strike a proper balance between protecting children’s interests when they are unable to do so themselves, and respecting their autonomy when they are. Since law is established on a strong presumption that persons older than a certain age are competent, whereas younger persons are not, it is decisive that a fixed age-limit for alleged competence is generally in accordance with children’s developmental stages. Furthermore, for pediatric patients and parents, availability of a reliable standard for assessing competence is important.

Children’s competence to consent, however, is currently not assessed in a standardized way. Moreover, neither the correlation between competence to give informed consent and age in children, nor which factors exactly contribute to children’s competence, have ever been systematically researched.

Former discussion on children’s competence to consent to medical issues has consistently been focused on normative concerns, which has impeded progress in clinical practices. The aim of this article is to offer recommendations for finding a way out of the impasse by means of identifying the gaps in knowledge about children’s competence issues and by suggesting options for dealing with the obstacles in empirical research. Empirical research outcomes are needed to make recommendations for optimizing policies, in order to do justice to the capacities and challenges children face when deciding about medical treatment and clinical research options.

## Discussion

### Normative aspects

History shows different perspectives on dealing with children’s competence issues. The enormity of the abuse in human experimentation in World War II led to the emergence of patients’ rights in medical decision-making [3]. In the ensuing years, competency issues involving children in clinical research proved especially problematic. The possibility of exposing children to the risk of harm was an ever present concern. But excluding them from research was not an option as biomedical research did prove successful: many examples showed that the mortality rate in children was drastically reduced [3]. To ensure research participation of children, while warranting their safety, specific pediatric regulations and guidelines were put in place. Nevertheless, until the nineties children kept being excluded from trials just to safeguard their vulnerability [4]. Developments regarding children’s competence in the treatment setting currently show that autonomous rights to self-determination have been extended and are now more variably assigned than before [5].

In law, competence has traditionally been associated with age [6]. Generally the law presumes that certain persons (i.e., adults) are competent, whereas others (children) are not. The statutory age of majority is commonly set at 18 years, although there are exceptions to this rule. Normally, young children under the age of 12 have no formal right to be involved in the informed consent process with their parents [7]. Differences exist between states and countries regarding the age at which children are deemed able to make competent decisions. In Europe, domestic law determines whether or not people are competent to consent to healthcare interventions [8]. In some countries autonomous decision-making is deemed legal at age 18, while in other countries minors are allowed to take healthcare decisions from a fixed age below legal majority, e.g., 14 years in Portugal and 15 years in Denmark [8]. A more flexible system exists in most Canadian provinces and Switzerland where the competence of children to consent is evaluated on a case-by-case basis [8]. In the United States statutes often specify various minimum ages (usually 12, 14, or 16 years) for independent consent by children for specific types of treatment [9]. In the United States regulations for clinical research state that some children under the age of 18 might be able to give their assent, meaning an affirmative agreement, but the institutional review board may still waive the assent requirement [10].

Obviously there is no international consensus on the exact age limit for presuming competence to consent in children [11]. To some extent, age limits seem arbitrary and ineffective as there are individuals above the limit who are deemed incompetent and individuals below the limit who are deemed competent. Though age limits are practicable, they may only serve their goal if they are generally in accordance. While statutory age limits have obviously taken into consideration the welfare of both society and the child, there is no clear empirical evidence regarding the competence of children’s age groups.

### Developmental aspects

Elementary school children face cognitive limitations; they lack the broad-based knowledge adults possess and sometimes have trouble applying their cognitive skills to a larger problem-solving process. They may view the world in concrete terms and cannot reason maturely about abstract and hypothetical problems [3, 12]. In adolescence, biological, cognitive, and social development progress and the brain undergoes substantial change with an increase in efficiency of brain functioning. New cognitive skills are acquired, referred to by Piaget as hypothetico-deductive reasoning: the ability to think of hypothetical solutions and to formulate a systematic plan for deducing which of these solutions is correct [12, 13]. Social-cognitive changes lead to increased maturity in reasoning about moral issues as well [14]. In middle adolescence (15 to 17) a strong development in metacognitive understanding emerges, including knowledge of one's own qualities, characteristics, and limitations with regard to decision-making [15]. Even with these advances, certain cognitive limitations remain, mostly involving inconsistent application of recently acquired cognitive abilities. Differences in decision-making between adolescents and adults have been found in the ability to act or think responsible, the ability to restrain impulsiveness, and the ability to place a given decision in a larger temporal context [16]. Data suggest that the adolescent brain still differs significantly from the adult brain, not least because the frontal lobes that are essential for effective executive functions mature later in children than they do in adults [17]. Adolescents generally do not fully possess the capacity to appreciate the long-term consequences of their choices until the age of 21 [17].

Apart from cognitive abilities, competence in children is thought to be related to life experience: children who have personal experiences with illness may show greater insight and understanding than children of comparable age who lack this experience [11, 18, 19].

Furthermore, as children grow up they are, to a greater extent than adults, dependent on other people, especially their caretakers or parents [12, 14, 20]. Children may be more obedient to parents and healthcare professionals because of their need for approval or fear of rebuke from authority figures [6, 9, 14, 21]. It is postulated that the quality of the relationship between the parent and the child and the doctor and patient is highly influential on the child’s ability to make well-informed decisions [22]. An authoritative parenting style which includes direction-giving and limit-setting is positively correlated with an adolescent’s developing capacity for autonomous decision-making [17]. Various authors argue that early adolescents (10 to 14) are more susceptible to peer influences than at any other age [16], which may hinder their ability to make thoughtful decisions [15].

### Empirical data

Extensive empirical research data on children’s decision-making competence are lacking. So far, only two studies on children’s competence to consent that comprised all four criteria have been conducted. Turrell and colleagues, using MacArthur Competence Assessment Tool for Treatment (MacCAT-T), conducted a comparative study on competence to consent in adolescents with anorexia nervosa and in adolescents considered healthy (in medicine known as healthy controls); that is, uncompromised by any disorders or illness, and found group differences: adolescents with anorexia nervosa tend to experience more problems in reasoning about treatment than healthy controls [23]. Koelch and colleagues examined the MacArthur Competence Assessment Tool for Clinical Research (MacCAT-CR) on a small sample of children and adolescents aged 7 to 12 diagnosed with Attention Deficit Hyperactivity Disorder and concluded that the tool was feasible and offered a detailed assessment, recommending further research on the validity of the tool [24]. Other studies directed at assessing children’s competence in medical decision-making vary widely; while often making use of both hypothetical and actual decision-making scenarios, these studies mostly measure only one dimension of competence, disregarding validity and reliability altogether [21].

### Practical barriers

Some authors consider the limited application of competence assessment in research a direct result from the lack of an operationalization of children’s decision-making competence [21]. Well-elaborated checklists that offer guidelines for good practice do exist [7, 19]; however, the last one was written half a decade ago and systematic research data to underpin these guidelines are not available. Reliability of a standard for assessing competence is important for health care professionals as well as pediatric patients and parents.

There is no consensus on which age spans to study, due to differences in local regulations. Decision-making situations that ask for data on children’s competence may concern: complex medical situations in exceptional cases, and more routine-like medical situations in the general pediatric population. However, it is not yet established how to incorporate the varying levels of risk and complexity of the decision into the assessment. One possible way to deal with the issue is to require a higher level of competence to consent for a decision with a higher potential risk and to require a higher level of competence to refuse for a decision with higher benefit [19]. Nevertheless, the level of risk is not yet well defined or quantifiable [11, 14].

Furthermore, it has been stated that consent to participation in research must be a more stringent process than consent to treatment, not necessarily because of higher levels of risk but because the research participants are asked to help improve general health care and thus do not profit individually [25]. However, there are no data yet to justify the supposed differences between children’s competence in the research and treatment context.

In addition, the discussion is ongoing on the dichotomous versus dimensional model of competence assessment. Buchanan and Brock [26] state that decision-making capacity is a matter of minor differences (gradual model) and competence is either present or not (threshold model). The gradual model may be more consistent with pediatric clinical practice, although a particular situation often requires a definitive assessment of competence. Some assessment instruments define decision-making capacity as the sum of different abilities, although we do not know whether these can be added up, or whether a threshold can be based on statistical arguments [27].

Besides, children’s competence relies on optimal information provision that enhances their understanding. Techniques for improving this include using clearly worded information tailored to their comprehension level. Decision-making can be facilitated by breaking the process down into smaller but linked choices. Communication difficulties can be overcome by innovative and age-appropriate techniques to convey information [12, 19]. When assessing children’s competence to consent, the quality and relevance of information must be optimized.

Although the need to evaluate competence to consent to treatment and clinical research has gained increasing attention, no consensus has been reached on how to assess it. The current situation has been referred to as a “hodgepodge of practices” [28]: there is no gold standard and no hard empirical data [14]. In the last two decades numerous tools have been developed to assess competence in adults. To name but a few: the Competency Questionnaire, the Hopkins Competency Assessment Test, the MacCAT-T and MacCAT-CR and the Structured Interview for Competency and Assessment Testing and Ranking Inventory [28]. Of all these, the MacCAT tools have ranked the highest and are considered the best choice for measuring capacity to consent to treatment and clinical research in adults [28]. Nonetheless, assessing competence to consent in children, even with adequate tools such as the MacCAT, is still lacking. As described above, only one study confirmed feasibility of the MacCAT-CR in children [24] and MacCAT-T has only been applied in adolescents [23].

### Directions

Clinical research on children’s competence to consent so far has been hampered by the absence of a guiding theory or framework with which to formulate hypotheses and interpret results. In discussions, a consensus definition of the clinical concept of children’s competence may be unreachable when taking into consideration the many questions under debate. Therefore setting out a research agenda regarding children’s competence to consent has not yet been accomplished.

For professionals it might be more profitable to find an approach in empirical research than to be entangled in substantial theoretical debate with no solution in sight. In adults, systematic studies using MacCAT-CR were conducted in populations of mentally compromised patients [29, 30]. Although the researchers faced comparable problems as described above, the research results contributed to further establishment of reliability and validity of a standardized competence assessment tool. Furthermore, results offered insight into the competencies of a given population regarding a given medical decision. Although the complexity of the clinical concept of children’s competence implies that competence assessment methods proposed by clinical researchers should not be expected to do full justice to all legal and ethical facets, still standardized methods are indispensable and can enhance insight into the clinical concept of children’s competence.

Experience from the small amount of research on children’s and adults’ competence to consent so far permits several recommendations for addressing the dilemmas in future research. Absent a standard, a clear definition and operationalization of children’s competence is needed. For this, consulting adult literature can be a starting point. The range of abilities to be studied must include the four relevant criteria which, to our knowledge, cover the basic aspects of assessment: understanding, appreciation, reasoning and expressing a choice [31]. Previous research demonstrated that the MacCAT scales provide the best results [28–30]. Implementing such a structured multidimensional tool would make it possible to systematically assess the different dimensions of competence [32] and make outcomes comparable [21].

Information provision should be optimized in competence assessment studies. Observational methods (video-taping or direct observation) can help us gain more insight into the decision-making process in children and identify aspects of the communication that facilitate decision-making [21, 33]. Actual decision-making, instead of hypothetical decision-making, may better reflect the way children and parents behave in real-world settings. More ethnical diversity in research samples is needed in order to increase generalizibility [21].

As children’s competence is likely to reflect a dynamic construct, it is important that the research design allows for these changes to be measured. The decision-making context for treatment and research is fundamentally different in many ways, but it is not clear if research and treatment situations elicit similar or different decision-making processes. Findings from studies that examine research decisions may not be generalizable to treatment decisions and may need different assessment methods, but the situations should be compared regardless.

Next to the legal requirements for competence that emphasize cognitive components such as understanding and reasoning ability, other important contextual variables that may influence children’s competence have not been very visible in research on competence so far and need more attention. The dependence of children on parents and caretakers [12, 14, 20] and the shift of parent orientation to peer orientation in adolescence [34] should be assessed.

The appropriate age span to be studied should cover ages at both the lower end as well as at the upper end of varying statutes and jurisdictions, leading to a study population of children from 7 to 18 years of age.

Different types of medical decisions must be studied in order to obtain data covering high risk, low risk, high complex, and low complex decisions. Although the level of risk and complexity are currently not quantifiable [11, 14], empirical research seems the most effective way to gather data and evaluate the relative contribution of these factors.

Considering that children’s personal experiences with illness might enhance their competence to consent [11, 18, 19, 21], there is good reason to assess the duration of the illness and the experience of the child with the healthcare system.

Two studies suggested a positive relationship between intelligence and competence, as measured by the Wechsler Intelligence Scale for Children [21]. Because cognitive capacities may play an important role in decision-making competence, assessment by an intelligence test should be included.

## Summary

The discussion on children’s competence to consent to treatment and clinical research has made little advancement the last decade. The ongoing debate involves many normative and developmental aspects and has not yielded progress in practical implementation. The empirical approach offers an opportunity to give direction to the debate and may lead to a clear research agenda.

Recommendations for future research include: studying a standardized assessment instrument covering all four relevant dimensions of competence; including a study population of children covering the full age range of 7 to 18 years; improving information provision; and assessing relevant contextual data. Research data are needed to underpin theories and guidelines and advance regulations concerning children’s decision-making competence in the medical context.

## Abbreviations

MacCAT-CR: MacArthur competence assessment tool for clinical research
MacCAT-T: MacArthur competence assessment tool for treatment.
